# Supplementary material for: Influence of LINE-Assisted Provision of Information about Human Papillomavirus and Cervical Cancer Prevention on HPV Vaccine Intention: A Randomized Controlled Trial
Source: Vaccines (Basel). 2022 Nov 24;10(12):2005. doi: 10.3390/vaccines10122005 (PMC9787927; doi:10.3390/vaccines10122005)
Supplement: Supplementary file 1 [file vaccines-10-02005-s001.zip › Supplymentaly figure.pdf]

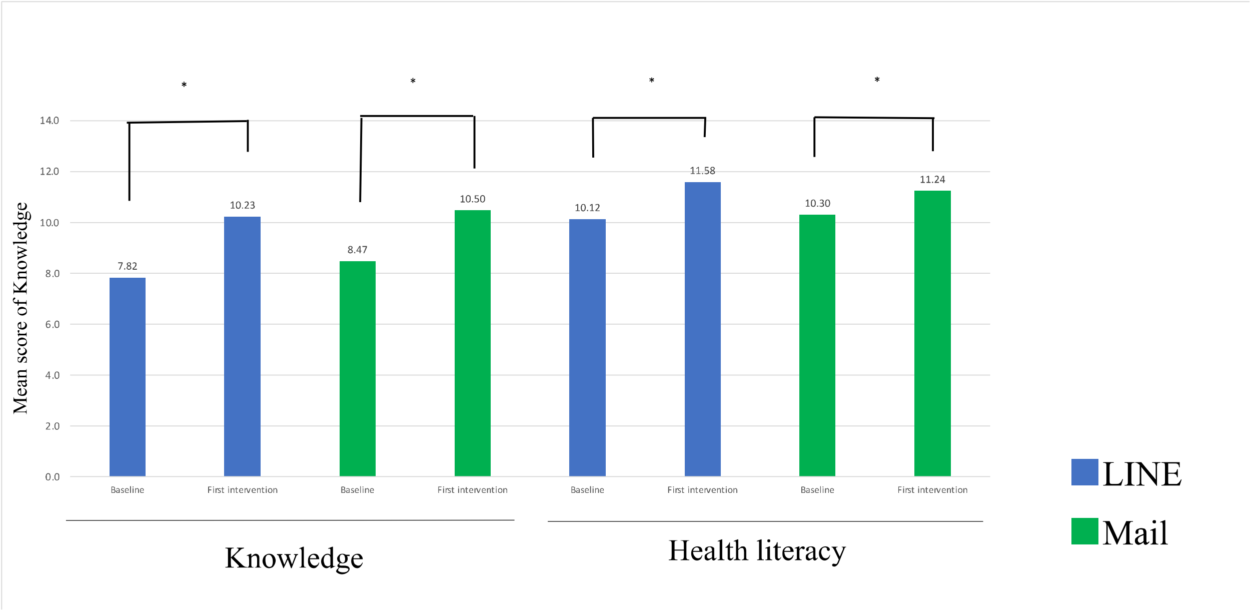

Figure S1. Comparison of HPV knowledge and Health literacy between baseline and the first intervention.

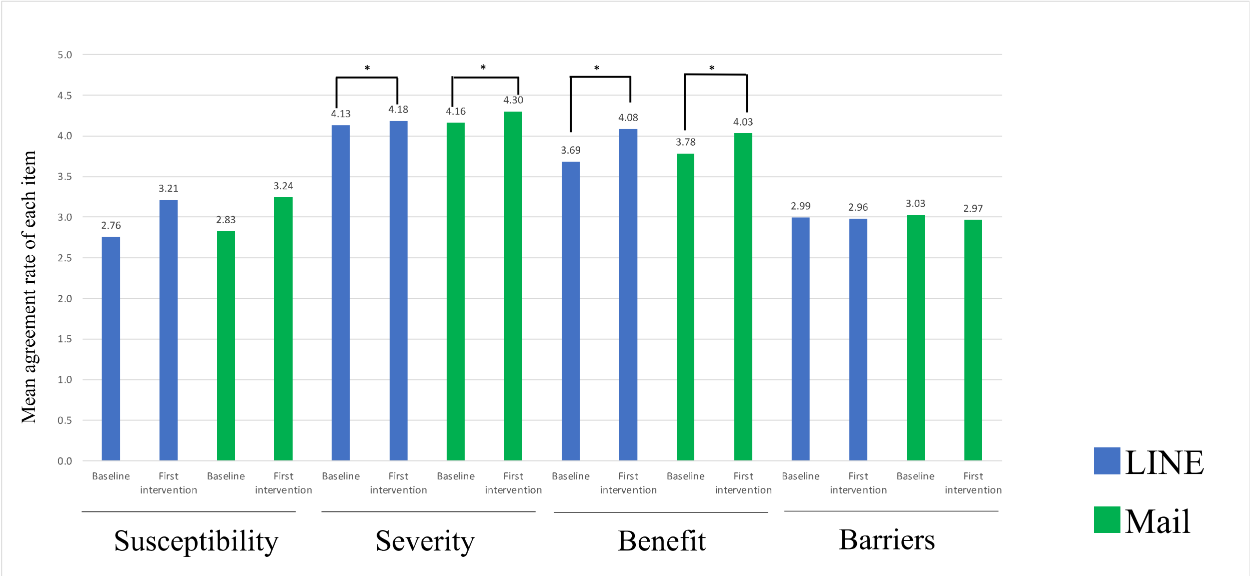

Figure S2. Comparison of Health belief models between baseline and the first intervention.

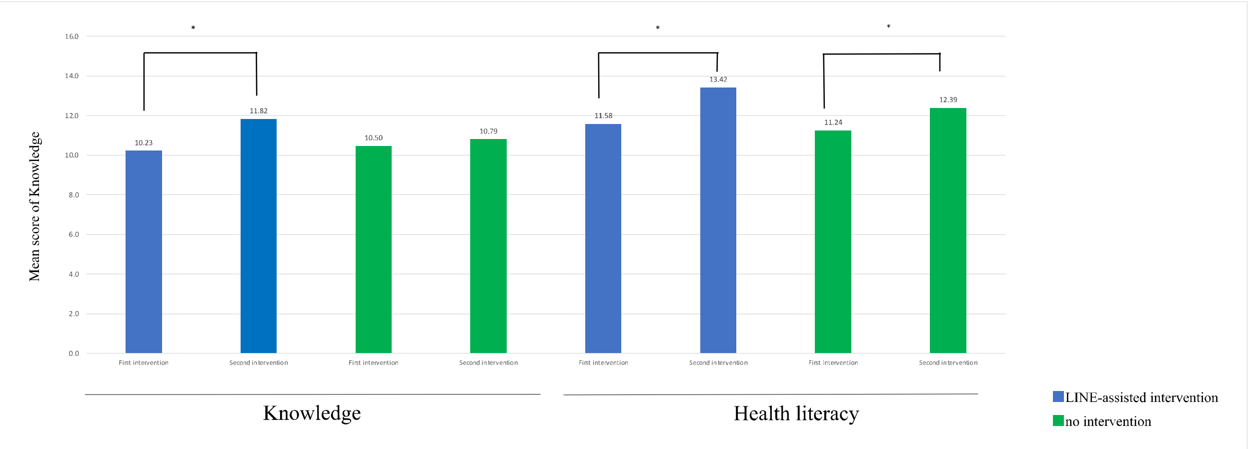

Figure S3. Comparison of HPV knowledge and Health literacy between the first and second intervention.

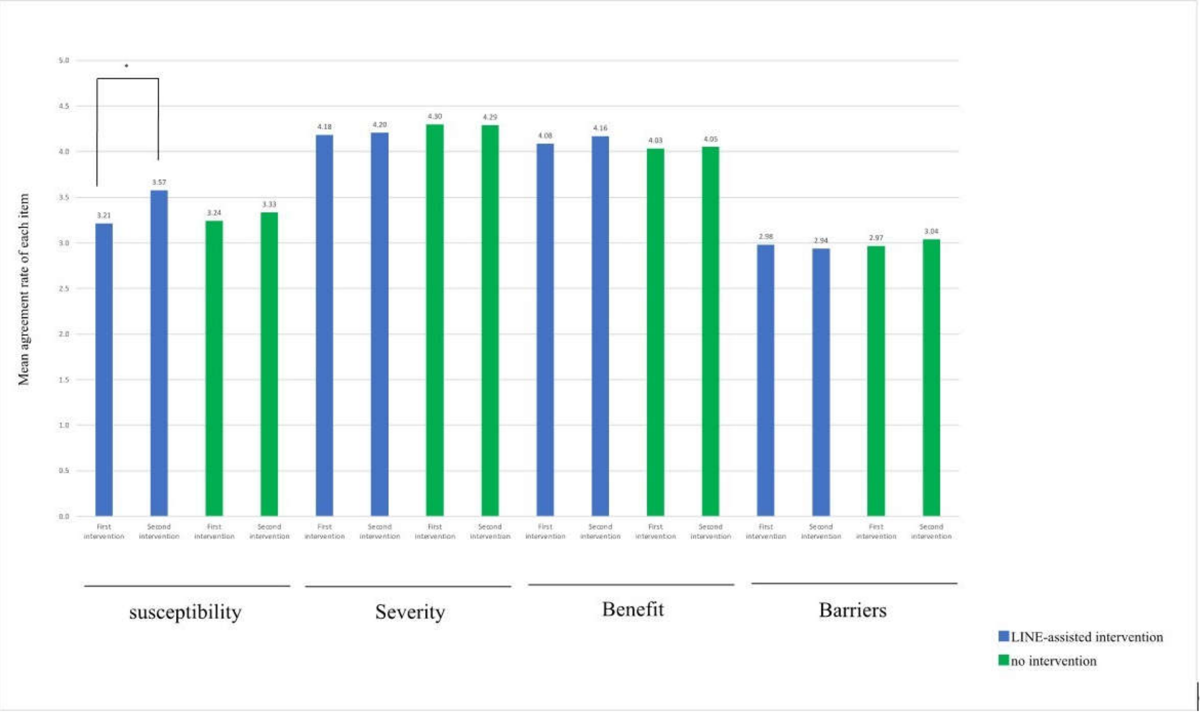

Figure S4. Comparison of Health belief models between the first and second intervention.
