# Supplementary material for: Influence of LINE-Assisted Provision of Information about Human Papillomavirus and Cervical Cancer Prevention on HPV Vaccine Intention: A Randomized Controlled Trial
Source: Vaccines (Basel). 2022 Nov 24;10(12):2005. doi: 10.3390/vaccines10122005 (PMC9787927; doi:10.3390/vaccines10122005)
Supplement: Supplementary file 1 [file vaccines-10-02005-s001.zip › Supplymentaly table.pdf]

Table S1. List of the questions in Google-form questionnaire.

Items		Questions	Correct answer
Characteristics			
Sex			
Age			
University			
Faculty		(medicine, nursing, science technology, or other)	
Smoking		(current, ever, never)	
Drinking		(everyday, a few times a week, seldom, never)	
Sleeping			
Exercise		exercise volume and intensity	
Breakfast		frequency of breakfast per week	
Reasons for not vaccinating			
		(concerns of side-effect, parents opposition, I don't know where to get vaccinated, expensive, sexually inactive, feel troublesome, I won't get infected, friends not vaccinated, only checkup is enough, schools do not recommend, afraid of get infected COVID-19, others)	
HPV vaccine intention			
		At what time do you intend to receive the HPV vaccine?	
		(Immediately, within six months, within a year, within three years, no intention to receive a vaccine, and not sure.)	
HPV knowledge		Questions (options)	
	1	HPV is a human immunodeficiency virus.	incorrect
	2	There are four types of HPV.	incorrect
	3	HPV causes cancer of the uterine body.	incorrect
	4	HPV cannot be transmitted by a single sexual intercourse.	incorrect
	5	HPV can only be transmitted by males, not by females.	incorrect
	6	Types 6 and 11 cause more than two-thirds of all cervical cancer.	correct
	7	Cervical cancer can be prevented by using vaccines.	correct
	8	Cervical cancer screening is done by cytology.	correct
	9	The eligible age for the HPV vaccine to be taken at public expense is up to 35 years old in Japan.	incorrect
	10	Cervical cancer is caused by persistent infection with high-risk HPV.	correct
		The options to these 10 questions were correct, incorrect, or do not know.	
	11	What do you think is the screening rate for cervical cancer in Japan?	
		(1. 80% or more, 2. 60-80%, 3. 40-60%, 4. 20-40%, 5. 20% or less)	4
	12	In some foreign countries, HPV vaccination is also given to males.	
		(1. correct, 2. do not know, 3. incorrect)	1
	13	What percentage of females with a history of sexually transmitted infections do you think will experience HPV infection by the time they are 50 years old?	
		(1. 80% or more, 2. 60-80%, 3. 40-60%, 4. 20-40%, 5. 20% or less)	2
	14	Which types of HPV are less likely to cause cancer?	

		(1. type 16, 2. type 18, 3. type 52, 4. type 31, 5. type 2)	5
	15	What is the leading cause of cancer death among females in their 20s and 30s?	
		(1. lung, 2. stomach, 3. liver, 4. uterus, 5. breast)	5
	16	It is necessary to have a regular checkup for cervical cancer even if you have HPV vaccine.	
		(1. correct, 2. do not know, 3. incorrect)	1
	17	How effective do you think the cervical cancer vaccination is in preventing cervical cancer?	
		(1. 100%, 2. 80-99%, 3. 60-70%, 4. 50% or less)	2
	18	How often do you think serious adverse reactions will occur due to vaccination compared to flu vaccine?	
		(1. Low, 2. About the same, 3. Somewhat high, 4. Extremely high)	2 or 3
	19	What do you think is the probability for the occurrence of cervical cancer in the lifetime of a female?	
		(1. one per two persons, 2. one per 12 persons, 3. one in 75 persons, 4. one in 155 persons)	3
	20	Which of the following do you think is used to confirm the diagnosis of cervical cancer?	
		(1. cytology, 2. hemolysis, 3. HPV virus type, 4. colposcopy (histology), 5. Pap smear)	4
Health literacy			
	1	Are you able to collect information related to the HPV vaccine and cervical cancer screening from various sources, such as newspapers, books, TV, and the Internet?	
	2	Are you able to select the information that one wants from a large amount of information related to the HPV vaccine and cervical cancer screening?	
	3	Are you able to consider the credibility of the information related to the HPV vaccine and cervical cancer screening?	
	4	Are you able to understand and communicate information related to the HPV vaccine and cervical cancer screening to others?	
	5	Are you able to decide on plans and actions to improve health based on the information about the HPV vaccine and cervical cancer screening?	
		The options to these questions were very easy, somewhat easy, intermediate, somewhat difficult, very difficult, or do not know/does not apply.	
Health beliefs			
Perceived susceptibility	1	I regard myself as susceptible to HPV infection.	
Perceived severity	2	I believe it would have potentially serious consequences if I get cervical cancer.	
	3	Cervical cancer is a life-threatening disease.	
Perceived benefit	4	Vaccination would reduce the susceptibility or severity or lead to other positive outcomes.	
Perceived barriers	5	The price of the vaccine is too expensive.	
	6	I am worried about the side effects of the vaccine.	
	7	I am too busy to go for the vaccination.	
	8	I do not know where I can get the vaccine.	
	9	Three doses of vaccination is too much trouble.	
	10	My parents do not agree with the vaccination.	

Table S2. Comparison of correct percentage of the detailed HPV knowledge items after the first and second intervention.

	Baseline (N = 357)			First intervention assessment (n = 295)			Second intervention assessent (n = 278)		
	Total	LINE (n = 178, 50%)	Mail (n = 179, 50%)	LINE (n = 146, 49%)	Mail (n = 149, 51%)	OR (95% CI)*	LINE-assisted intervention (n = 142, 51%)	No intervention (n = 136, 49%)	OR (95% CI)*
	N (%)	N (%)	N (%)	N (%)	N (%)		N (%)	N (%)	
HPV stands for human immunodeficiency virus.									
	153 (43)	68 (39)	85 (47)	67 (47)	71 (48)		70 (49)	65 (48)	
Per protocol						0.96 (0.61-1.52)			1.06 (0.66-1.69)
Intention to treat						1.01 (0.62-1.64)			1.05 (0.66-1.66)
There are four types of HPV.									
	56 (16)	27 (15)	29 (16)	57 (40)	57 (39)		65 (46)	54 (40)	
Per protocol						1.04 (0.65-1.67)			1.26 (0.78-2.03)
Intention to treat						1.09 (0.67-1.79)			1.31 (0.83-2.08)
HPV causes uterine cancer.									
	88 (25)	40 (23)	48 (27)	54 (38)	50 (34)		54 (38)	52 (39)	
Per protocol						1.22 (0.75-1.98)			0.98 (0.60-1.59)
Intention to treat						1.41 (0.88-2.26)			1.03 (0.66-1.59)
HPV cannot be transmitted through a single sexual encounter.									
	272 (77)	133 (76)	139 (78)	131 (90)	135 (91)		129 (91)	122 (90)	
Per protocol						0.97 (0.44-2.10)			1.24 (0.55-2.78)
Intention to treat						0.95 (0.51-1.78)			1.32 (0.74-2.32)
HPV can only be transmitted to women, not to men.									
	223 (63)	102 (58)	121 (68)	123 (86)	134 (90)		127 (91)	121 (90)	
Per protocol						0.69 (0.34-1.42)			1.12 (0.51-2.48)
Intention to treat						0.76 (0.42-1.38)			1.25 (0.71-2.21)
Types 6 and 11 cause more than two-thirds of all cervical cancers.									
	40 (11)	15 (9)	25 (14)	31 (22)	33 (22)		52 (37)	38 (28)	
Per protocol						0.95 (0.55-1.65)			1.50 (0.90-2.49)
Intention to treat						0.97 (0.54-1.75)			1.31 (0.78-2.20)
Cervical cancer can be prevented by vaccines.									
	256 (72)	126 (72)	130 (73)	127 (88)	134 (90)		132 (93)	128 (95)	
Per protocol						0.84 (0.40-1.74)			0.73 (0.27-1.97)
Intention to treat						0.79 (0.45-1.39)			0.96 (0.44-2.10)
Cervical cancer screening is done by cytology.									

	115 (32)	59 (34)	56 (31)	84 (58)	94 (64)		107 (75)	85 (63)	
Per protocol						0.77 (0.48-1.24)			1.78 (1.07-3.00)
Intention to treat						0.85 (0.51-1.40)			1.89 (1.13-3.16)
Cervical cancer can be prevented by vaccination.									
	50 (14)	17 (10)	33 (18)	62 (43)	68 (46)		75 (53)	52 (39)	
Per protocol						0.90 (0.57-1.43)			1.78 (1.11-2.87)
Intention to treat						0.93 (0.61-1.44)			1.68 (1.04-2.72)
Cervical cancer is caused by persistent infection with high-risk HPV.									
	122 (34)	57 (33)	65 (36)	77 (53)	84 (56)		97 (68)	72 (53)	
Per protocol						0.88 (0.56-1.39)			1.88 (1.51-3.07)
Intention to treat						0.91 (0.58-1.41)			2.02 (1.28-3.19)
What do you think is the cervical cancer screening uptake rate in Japan?									
	131 (37)	61 (34)	70 (39)	49 (34)	59 (40)		63 (44)	56 (41)	
Per protocol						0.78 (0.49-1.23)			1.13 (0.70-1.81)
Intention to treat						0.71 (0.44-1.15)			1.08 (0.66-1.75)
In some foreign countries, HPV vaccination is also offered to men.									
	196 (55)	93 (53)	103 (58)	110 (76)	115 (78)		122 (86)	106 (79)	
Per protocol						0.88 (0.51-1.53)			1.66 (0.89-3.10)
Intention to treat						0.85 (0.47-1.55)			1.42 (0.84-2.36)
Approximately what percentage of women who have had sexually transmitted diseases do you think will experience HPV infection by the age of 50?									
	45 (13)	18 (10)	27 (15)	35 (24)	43 (29)		37 (26)	38 (28)	
Per protocol						0.79 (0.47-1.33)			0.91 (0.54-1.55)
Intention to treat						0.87 (0.53-1.41)			0.96 (0.55-1.66)
Which of the following types of HPV is less likely to cause cancer?									
	94 (28)	42 (25)	52 (32)	45 (31)	51 (35)		47 (34)	40 (30)	
Per protocol						0.86 (0.57-1.40)			1.20 (0.72-2.00)
Intention to treat						0.87 (0.54-1.42)			1.07 (0.68-1.70)
Which of the following is the most common cause of cancer death among women in their 20s and 30s?									
	144 (41)	74 (42)	70 (39)	45 (31)	48 (32)		51 (36)	42 (31)	
Per protocol						0.95 (0.58-1.55)			1.24 (0.75-2.04)
Intention to treat						1.04 (0.65-1.67)			1.32 (0.82-2.11)
Even if you have had the HPV vaccine, you still need to have regular cervical cancer screenings.									
	313 (88)	154 (87)	159 (90)	132 (91)	138 (93)		131 (94)	125 (93)	
Per protocol						0.74 (0.31-1.73)			1.05 (0.40-2.72)
Intention to treat						0.87 (0.43-1.75)			1.32 (0.57-3.06)
By what percentage do you think the cervical cancer vaccination is effective in preventing cervical cancer?									

	190 (53)	100 (56)	90 (51)	102 (70)	98 (66)		106 (75)	91 (67)	
Per protocol						1.23 (0.75-2.01)			1.46 (0.84-2.46)
Intention to treat						1.19 (0.74-1.93)			1.55 (0.95-2.52)
How often do you think serious adverse reactions occur due to vaccination compared to influenza or Japanese encephalitis vaccination?									
	258 (73)	129 (72)	129 (73)	96 (66)	87 (88)		93 (65)	82 (61)	
Per protocol						1.39 (0.87-2.24)			1.23 (0.75-2.00)
Intention to treat						1.49 (0.94-2.34)			1.36 (0.83-2.23)
What do you think is the probability that a woman will develop cervical cancer in her lifetime?									
	105 (30)	48 (27)	57 (32)	36 (25)	35 (24)		86 (61)	76 (56)	
Per protocol						1.06 (0.62-1.80)			1.21 (0.75-1.96)
Intention to treat						1.04 (0.64-1.71)			1.30 (0.82-2.07)
Which of the following do you think is the definitive diagnosis of cervical cancer?									
	56 (16)	28 (16)	28 (16)	30 (21)	29 (19)		32 (23)	28 (21)	
Per protocol						1.09 (0.62-1.93)			1.11 (0.63-1.97)
Intention to treat						1.21 (0.71-2.06)			1.07 (0.63-1.79)

* Logistic regression model stratified by sex

Numbers in each category that do not reach total numbers indicate missing data.

Table S3. Perception of Health belief models after the first and second intervention.

Characteristics	Baseline (n = 357)			First intervention assessment (n = 295)			Second intervention assesent (n = 278)		
	Total	LINE (n = 178, 50%)	Mail (n = 179, 50%)	LINE (n = 146, 49%)	Mail (n = 149, 51%)	OR (95% CI)*	LINE-assisted intervention (n = 142, 51%)	No intervention (n = 136, 49%)	OR (95% CI)*
	n (%)	n (%)	n (%)	n (%)	n (%)		n (%)	n (%)	
Perceived susceptibility									
There is a possibility that I may be exposed to HPV in the future.									
	151 (42)	68 (38)	83 (46)	79 (54)	81 (55)		90 (63)	79 (59)	
Per protocol						0.97 (0.61-1.54)			1.25 (0.76-2.04)
Intention to treat						1.02 (0.65-1.62)			1.32 (0.86-2.04)
Perceived severity									
Cervical cancer is a scary and life-threatening disease									
	276 (78)	137 (77)	139 (78)	110 (76)	122 (82)		121 (85)	111 (82)	
Per protocol						0.69 (0.39-1.23)			1.25 (0.66-2.36)
Intention to treat						0.72 (0.42-1.22)			1.22 (0.75-1.98)
Cervical cancer can have serious consequences in the future									
	294 (83)	140 (79)	154 (86)	118 (81)	123 (83)		113 (80)	119 (88)	
Per protocol						0.89 (0.49-1.62)			0.53 (0.27-1.01)
Intention to treat						0.89 (0.52-1.52)			0.80 (0.49-1.30)
Perceived benefit									
Vaccination can prevent cervical cancer.									
	235 (66)	112 (64)	123 (69)	113 (78)	115 (78)		112 (79)	106 (79)	
Per protocol						1.02 (0.59-1.77)			1.02 (0.58-1.81)
Intention to treat						1.00 (0.62-1.62)			1.11 (0.67-1.84)
Perceived barriers									
Vaccination can prevent cervical cancer) (Vaccine price is too high (usually 15,000 yen per dose, 3 doses).									
	241 (68)	115 (65)	126 (70)	105 (72)	114 (77)		101 (71)	95 (70)	
Per protocol						0.77 (0.46-1.32)			1.05 (0.62-1.76)
Intention to treat						0.84 (0.52-1.34)			1.28 (0.81-2.04)
I'm worried about the side effects of the vaccine.									
	195 (55)	106 (60)	89 (50)	73 (50)	54 (36)		62 (44)	56 (41)	
Per protocol						1.78 (1.10-2.86)			1.11 (0.68-1.80)
Intention to treat						1.74 (1.08-2.83)			1.13 (0.71-1.79)
I'm too busy to go get vaccinated.									
	145 (41)	64 (36)	81 (46)	55 (38)	65 (44)		49 (35)	60 (45)	
Per protocol						0.77 (0.48-1.23)			0.67 (0.41-1.08)
Intention to treat						0.78 (0.50-1.23)			0.67 (0.42-1.06)
I don't know where I can get the vaccine.									

	201 (56)	90 (51)	111 (62)	68 (47)	84 (57)		53 (37)	67 (50)	
Per protocol						0.67 (0.42-1.06)			0.61 (0.38-0.98)
Intention to treat						0.67 (0.43-1.02)			0.67 (0.42-1.06)
Three vaccinations is too much trouble									
	214 (60)	110 (62)	104 (58)	97 (67)	81 (55)		85 (60)	84 (62)	
Per protocol						1.66 (1.03-2.67)			0.90 (0.56-1.46)
Intention to treat						1.62 (1.01-2.59)			1.01 (0.66-1.56)
My parents don't agree with the vaccination									
	66 (19)	36 (20)	30 (17)	24 (17)	20 (14)		27 (19)	18 (13)	
Per protocol						1.26 (0.66-2.41)			1.60 (0.83-3.11)
Intention to treat						1.20 (0.65-2.24)			1.57 (0.87-2.83)

* Logistic regression model stratified by sex

Numbers in each category that do not reach total numbers indicate missing data.

Table S4. Comparison of HPV vaccine intention between baseline and the first intervention.

		HPV vaccine intention at the first intervention				
		Yes		No		p*
		N	%	N	%	
LINE group						
	HPV vaccine intention at Baseline					0.046
	Yes	47	80	12	20	
	No	24	29	58	71	
Mail group						
	HPV vaccine intention at Baseline					0.317
	Yes	44	68	21	32	
	No	15	18	67	82	

*Mc Nemar's test
